# PI-3K/Akt Pathway-Dependent Cyclin D1 Expression Is Responsible for Arsenite-Induced Human Keratinocyte Transformation

**DOI:** 10.1289/ehp.10403

**Published:** 2007-10-05

**Authors:** Weiming Ouyang, Wenjing Luo, Dongyun Zhang, Jinlong Jian, Qian Ma, Jingxia Li, Xianglin Shi, Jingyuan Chen, Jimin Gao, Chuanshu Huang

**Affiliations:** 1 Nelson Institute of Environmental Medicine, New York University School of Medicine, Tuxedo, New York, USA; 2 Department of Occupational and Environmental Health Sciences, Fourth Military Medical University, Xi’an, Shaanxi, People’s Republic of China; 3 Graduate Center for Toxicology, University of Kentucky, Lexington, Kentucky, USA; 4 Zhejiang Provincial Key Laboratory of Medical Genetics, School of Life Sciences, Wenzhou Medical College, Wenzhou, Zhejiang, People’s Republic of China

**Keywords:** Akt, arsenite, cyclin D1, human keratinocyte, PI-3K

## Abstract

**Background:**

Long-term exposure of arsenite leads to human skin cancer. However, the exact mechanisms of arsenite-induced human skin carcinogenesis remain to be defined.

**Objectives:**

In this study, we investigated the potential role of PI-3K/Akt/cyclin D1in the transformation of human keratinocytic cells upon arsenite exposure.

**Methods:**

We used the soft agar assay to evaluate the cell transformation activity of arsenite exposure and the nude mice xenograft model to determine the tumorigenesis of arsenite-induced transformed cells. We used the dominant negative mutant and gene knockdown approaches to elucidate the signaling pathway involved in this process.

**Results:**

Our results showed that repeated long-term exposure of HaCat cells to arsenite caused cell transformation, as indicated by anchorage-independent growth in soft agar. The tumorigenicity of these transformed cells was confirmed in nude mice. Treatment of cells with arsenite also induced significant activation of PI-3K and Akt, which was responsible for the anchorage-independent cell growth induced by arsenite exposure. Furthermore, our data also indicated that cyclin D1 is an important downstream molecule involved in PI-3K/Akt–mediated cell transformation upon arsenite exposure based on the facts that inhibition of cyclin D1 expression by dominant negative mutants of PI-3K, and Akt, or the knockdown of the cyclin D1 expression by its specific siRNA in the HaCat cells resulted in impairing of anchorage-independent growth of HaCat cells induced by arsenite.

**Conclusion:**

Our results demonstrate that PI-3K/Akt–mediated cyclin D1 expression is at least one key event implicated in the arsenite human skin carcinogenic effect.

Arsenite is a well-documented human carcinogen. Long-term exposure to inorganic arsenic from drinking water has been reported to induce various cancers (Centeno et al. 2002; Huang et al. 2004; Tseng et al. 1968; Yu et al. 2006). Chronic exposure to arsenite can lead to its accumulation in the skin and cause skin hyperpigmentation and hyperkeratosis (Centeno et al. 2002; Yu et al. 2006). This could in turn develop into skin cancers, including Bowen disease (carcinoma *in situ*), basal cell carcinoma (BCC), and squamous cell carcinoma (SCC) (Tseng et al. 1968; Yu et al. 2006).

Cancer development results from a synergism between genotoxic and nongenotoxic factors (Hecker 1987; Zoumpourlis et al. 2003). The former induces irreversible genetic alterations (tumor initiation), whereas the latter promotes tumor development by favoring the clone outgrowth of the genetically altered cells (tumor promotion) through activating cell survival and proliferation signal pathways and altering the machineries controlling cell proliferation and apoptosis. Previous studies have demonstrated that arsenite has a weak mutagen effect; therefore it is thought that its ability to activate signaling pathways leading to the alteration of gene expression responsible for cell growth may play an important role in its carcinogenic effect (Bernstam and Nriagu 2000). It has been demonstrated that signal pathways, including mitogen-activated protein kinases (MAPKs), activating factor 1 (AP-1) and nuclear factor kappa B (NF-κB), can be activated upon arsenite exposure and presumably contribute to arsenite-induced skin carcinogenic effect (Cooper et al. 2004; Huang et al. 2001, 2004). Phosphatidylinositol 3-kinase (PI-3K) comprises an 85-kDa regulatory subunit (p85) and a 110-kDa catalytic subunit (p110) and could be activated by multiple growth factors and cytokines (Cantley 2002; Vivanco and Sawyers 2002). Upon activation, PI-3K generates phosphatidylinositol-3,4,5-trisphosphate (PIP3), a lipid second messenger essential for the activation of protein kinase B (Akt) (Alessi et al. 1997; Toker and Cantley 1997). Akt in turn regulates various cellular functions such as apoptosis and proliferation (Alessi et al. 1997; Franke et al. 2003). PI-3K/Akt has been demonstrated to be an important signaling pathway for cell survival and growth, and it also plays a pivotal role in cell transformation and tumorigenesis (Huang et al. 1999; Li et al. 2005; Nicholson and Anderson 2002; Ouyang et al. 2005a; Samuels and Ericson 2006). The elevated expression or high phosphorylation of Akt could be observed in many tumor cells (Asanuma et al. 2005; Bae et al. 2006; Misra et al. 2006). Most recently, He et al. (2006) reported that PI-3K/Akt is related to the malignant transformation associated with acquired apoptotic resistance in human HaCaT keratinocytes induced by chronic UVA irradiation. Souza et al. (2001) have reported that PI-3K is required for the induction of endothelial nitric oxide synthesis (eNOS) by arsenite in human keratinocytes. Our previous studies have also shown that arsenite exposure is able to activate the PI-3K/Akt pathway and induce cyclin D1 expression in mouse epidermal Cl41 cells (Ouyang et al. 2006). In HaCat cells, the PI-3K/Akt/cyclin D1 cascade activation contributed to arsenite-induced proliferation (Ouyang et al. 2007b). Although hyperproliferation is correlated with cellular transformation in some cases (Chen et al. 2001), our previous findings clearly demonstrated that in Cl41 cells, epidermal growth factor (EGF)-induced transformation was impaired by disrupting PI3K/p85 expression; however, cell proliferation was not affected (Huang et al. 1996), which indicates that the transformation ability is not always paralleled with the accelerated proliferation rate. Therefore, we performed the present studies to investigate whether the PI-3K/Akt signal pathway is indeed implicated in arsenite-induced cell transformation through the induction of cyclin D1.

## Materials and Methods

### Cell culture and reagents

Spontaneously immortalized human keratinocytes, HaCat cell line, and their stable transfectants were cultured in monolayers at 37°C, 5% CO_2_ using Dulbecco’s modified Eagle’s medium (DMEM) containing 10% fetal bovine serum (FBS), 2 mM l-glutamine, and 25 μg gentamicin/mL. Normal human epidermal keratinocytes (NHEKs) were cultured in keratinocyte–SFM medium (Invitrogen Corp., Carlsbad, CA, USA) containing supplements (human epidermal growth factor, bovine pituitary extract; Invitrogen) and gentamycin (5 mg/mL; Sigma-Aldrich Corp., St. Louis, MO, USA). The cultures were detached with trypsin and transferred to new 75-cm^2^ culture flasks (Fisher Scientific Co., Pittsburgh, PA, USA) twice a week. FBS was purchased from Life Technologies, Inc.; DMEM was from Calbiochem (San Diego, CA, USA); sodium arsenite was purchased from Aldrich Chemical Co. Inc. (Milwaukee, WI, USA). The dominant-negative mutants of Akt (DN-Akt) and PI-3K (Δp85) were described in our previous studies (Huang et al. 1996; Li et al. 2004; Ouyang et al. 2006).

### Cyclin D1 small interference RNA construction

The specific small interference RNA (siRNA)–targeted human cyclin D1 was described before (Ouyang et al. 2005b). The target sequence was inserted into the pSuppressor vector and verified by DNA sequencing.

### Stable transfection

We transfected HaCat cells with DN-Akt and Δp85 or vector control plasmids using Lipofectamine 2000 reagent (Invitrogen Corp.) according to manufacturer’s instructions. Briefly, HaCat cells were cultured in a 6-well plate to 85–90% confluence. Five micrograms plasmid DNA, alone or in combination with pCMV-neo vector, were for co-transfection. DNA was mixed with 10 μL of Lipofectamine 2000 reagent and used to transfect each well in the absence of serum. After 6–8 hr, the medium was replaced with 10% FBS DMEM. Approximately 30–36 hr after the beginning of the transfection, the cells were detached with 0.033% trypsin, and cell suspensions were plated into 75-mL culture flasks and cultured for 24–28 days with G418 selection (800 μg/mL). Stable transfectants were established and cultured in G418-free DMEM for at least two passages before each experiment. HaCat cells were stably transfected with siCyclin D1 as established and identified in our published studies (Ouyang et al. 2005b).

### PI-3 kinase assay

We conducted the PI-3 kinase activity assay as described in our previous reports (Huang et al. 1996; Ouyang et al. 2006). Briefly, cells were cultured in monolayers in 100-mm dishes using normal culture medium. The medium was replaced with 0.1% FBS DMEM containing 2 mM l-glutamine and 25 μg gentamicin/mL after the cell density reached 70–80%. Forty-five hours later, we incubated the cells with fresh serum-free DMEM for 3–4 hr at 37°C. Arsenite was then added to the cell cultures for PI-3K induction. The cells were washed once with ice-cold PBS and lysed in 400 μL lysis buffer/plate [20 mM Tris (pH 8.0), 137 mM NaCl, 1 mM MgCl_2_, 10% glycerol, 1% NP-40, 1-mM DTT, 0.4 mM sodium ortho-vanadate, and 1 mM phenylmethylsulfonyl fluoride]. The lysates were centrifuged and the supernatants were incubated at 4°C with 40 μL agarose beads (conjugated with the anti-phosphotyrosine antibody Py20) overnight. Beads were washed twice with each of the following buffers: *a*) PBS with 1% NP-40, 1 mM DTT; *b*) 0.1 M Tris (pH 7.6), 0.5 M LiCl, l mM DTT; and *c*) 10 mM Tris (pH 7.6), 0.1 M NaCl, 1 mM DTT. Beads were incubated for 5 min on ice in 20 μL buffer 3, then 20 μL of 0.5 mg/mL phosphatidylinositol [sonicated previously in 50 mM HEPES (pH 7.6), 1 mM EGTA, 1 mM NaH_2_PO_4_] were added. After 5 min at room temperature, 10 μL of the reaction buffer were added [50 mM MgCl_2_, 100 mM HEPES (pH 7.6), 250 μM ATP containing 5 μCi γ-^32^P- ATP], and the beads were incubated for an additional 15 min. The reactions were stopped by the addition of 15 μL of 4 N HCl and 130 μL chloroform/methanol (1:1). After vortexing for 30 sec, the solutions, 30 μL from the phospholipid-containing chloroform phase were spotted onto thin-layer chromagraphy plates coated with silica gel H containing 1.3% potassium oxalate and 2 mM EDTA applied in H_2_O/methanol (3:2). The plates were heated at 110°C for at least 3 hr before use. The plates were then placed in tanks containing chloroform/methanol/ammonium hydroxide/H_2_O (600:470:20:113) for 40–50 min until the solvent reached the top of the plates. The plates were dried at room temperature and autoradiographed.

### Western blot analysis

We cultured HaCat cells and their transfectants (2 × 10^5^) in each well of 6-well plates to 70–80% confluence with normal culture medium. The cell culture medium was replaced with 0.1% FBS DMEM with 2 mM l-glutamine and 25 μg gentamicin and cultured for 43 hr. The cells were incubated in serum-free DMEM for 3–4 hr at 37°C. After exposure to arsenite, the cells were washed once with ice-cold PBS, then extracted with sodium dodecyl sulfate (SDS)-sample buffer. The cell extracts were separated on polyacrylamide–SDS gels, transferred, and probed with each of the antibodies against phosphor-specific Akt (Thr308), phosphor-specific Akt (Ser473), Akt, cyclin D1, and glyceraldehyde 3-phosphate dehydrogenase. The protein bands specifically bound to the primary antibodies were detected using an anti-rabbit IgG alkaline phosphatase-linked secondary antibody and an ECF (enhanced chemifluorescence) Western blot analysis system (Amersham Pharmacia Biotech, Piscataway, NJ, USA) (Ouyang et al. 2006).

### Cell proliferation assay

Confluent monolayers of HaCat cells were trypsinized, and 1 × 10^3^ of viable cells suspended in 100 μL DMEM supplemented with 10% FBS were added to each well of 96-well plates. The plates were incubated at 37°C in a humidified atmosphere of 5% CO_2_. Twelve hours later, we exposed the cells to arsenite for 5 days at the concentrations indicated. The exposed cells were lysed with 50 μL lysis buffer, and the proliferation of the cells was measured using CellTiter-Glo Luminescent Cell Viability Assay kit (Promega, Madison, WI, USA) with a luminometer (Wallac 1420 Victor2 multipliable counter system; Perkin-Elmer Life and Analytical Sciences, Inc., Waltham, MA, USA). The results are expressed as luciferase activity relative to control medium (proliferation index).

### Anchorage-independent growth

We cultured HaCat cells and their transfectants (1 × 10^5^) in each well of 6-well plates to 50–60% confluence with normal culture medium. The cells were treated with 2.5 μM arsenite for 3 days, then recovered in fresh medium for 1 day. After the repeated treatment with arsenite for 8 weeks, the cells were used for anchorage-independent growth assay, which was performed as described previously (Huang et al. 1999; Yan et al. 2006). Briefly, 2.5 mL of 0.5% agar in basal modified Eagle’s medium (BMEM) supplemented with 10% FBS was laid onto each well of 6-well tissue culture plates. We mixed 2 × 10^4^ HaCat cells with 2 mL of 0.5% agar BMEM and layered the cells on top of the 0.5% agar layer. The plates were incubated at 37°C in 5% CO_2_ for 3 weeks. We then scored the colonies with more than 16 cells.

### Tumorigenicity assays

We randomly divided six 5-week-old female nude mice into two experimental groups—medium control group and arsenite-treated group. Each nude mouse was injected sc in two spots with 2 × 10^6^ of cells in 100 μL of growth medium for each spot. The mice were sacrificed by CO_2_ asphyxiation 4 weeks after the inoculation, tumor dimensions were measured using calipers and tumor volume (cubic millimeters) was calculated using the following formula: 0.5236 (L × W × H) as described in previous studies (Brubaker et al. 2006; Jungwirth et al. 1997), where L is tumor length, W is width, and H is height (Ouyang et al. 2007a). Tumors were removed from mice, and fixed in 10% buffered formalin and embedded in paraffin; 5-μm sections were dehydrated and stained with hematoxylin and eosin (H&E).

### Statistical analysis

The significant difference between the treated and untreated groups was determined with the Student *t*-test. Results are expressed as mean ± SD.

## Results

### Repeated arsenite exposure led to transformation of HaCat cells

**Figure 1:**
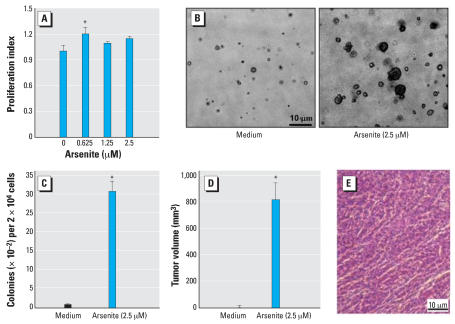
Induction of cell transformation by arsenite in human keratinocyte HaCat. (*A*) HaCat cells were exposed to various doses of arsenite for 5 days. The proliferation of the cells was measured using CellTiter-Glo Luminescent Cell Viability Assay kit with a luminometer. (*B*,*C*) HaCat cells were then repeatedly exposed to 2.5 μM arsenite twice a week for a total of 8 weeks as described in “Materials and Methods.” (*D*) 2 × 10^6^ of above cells were injected sc into each spot of 5-week-old female nude mice. Four weeks after the inoculation, the tumor dimensions were measured using calipers and tumor volume (mm^3^) was calculated. The data shown are from six tumors in three mice for each group. (*E*) Paraffin-embedded tumor xenografts were sectioned (4 μm) and subjected to H&E staining. *Significant increase compared with that of medium control (*p* < 0.05).

Human skin is a major target of environmental carcinogen arsenite. To elucidate the mechanism implicated in arsenite-induced human skin carcinogenic effect *in vitro*, we first evaluated the cytotoxicity of arsenite to HaCat cells with CellTiter-Glo Luminescent Cell Viability Assay kit. We found that exposure of HaCat cell to 0.625 μM arsenite caused a significant increase in cell proliferation (Figure 1A) and no inhibition of cell proliferation at doses lower than 2.5 μM arsenite (Figure 1A). Thus, we used 2.5 μM arsenite to treat human keratinocyte HaCat cells to establish a cell transformation model. HaCat cells were exposed repeatedly to 2.5 μM arsenite twice a week for 8 weeks, and the anchorage-independent growth capability of arsenite-treated HaCat cells was evaluated. Compared with the medium control, repeated arsenite exposure resulted in increased the anchorage-independent growth capacity of HaCat cells (Figure 1B, C). Those results indicate that arsenite-exposed HaCat cells obtain the ability of anchorage-independent growth for colony formation in soft agar. The tumor characteristic of the transformed cells was further confirmed in nude mice. As shown in Figure 1D, injection of arsenite long-term exposed Hacat cells into nude mouse caused observable tumor formation (tumor volumes 786 ± 126, *n* = 6) compared with that of long-term culture HaCat cells (0 ± 0, *n* = 6). H&E staining also revealed a tumor formation in the arsenite long-term exposed Hacat cells (Figure 1E). On the basis of these results, we anticipate that repeated exposure of HaCat cells to arsenite could cause malignant transformation.

### The PI-3K/Akt pathway is required for arsenite-induced transformation of HaCat cells

**Figure 2:**
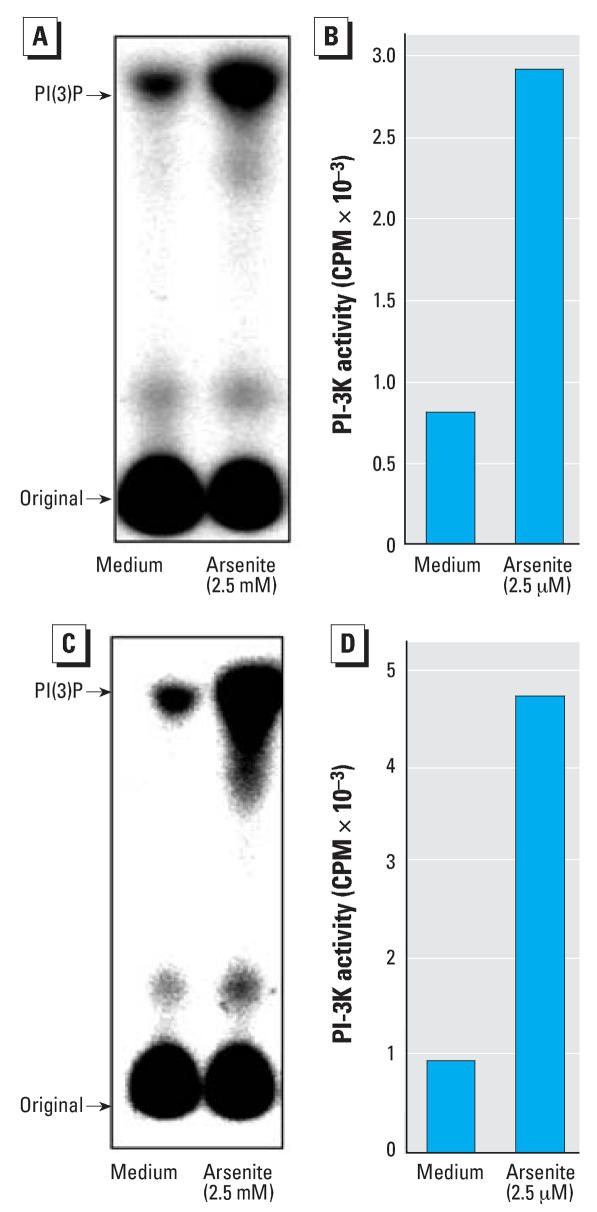
PI-3K activation induced by arsenite in both HaCat and NHEKs. (*A*,*B*) HaCat cells with 70–80% confluence were exposed to 2.5 μM arsenite for 30 min, and the cells were harvested. The PI-3K activity was determined as described in “Materials and Methods.” The results were shown as an autoradiograph (*A*) and schematic diagram of the PI-3K product PI(3)P from the PI-3K assay spot (CPM) (*B*). PI-3K activity by arsenite was determined in primary cultured normal human epidermal keratinocytes (*C*) and schematic diagram of the PI-3K product PI(3)P from the PI-3K assay spot (CPM) (*D*). The data shown represent one of three independent experiments.

Our previous studies have shown that PI-3K is essential for Cl41 cells obtaining anchorage-independent growth capacity in TPA (12-*O*-tetradecanoylphorbol-13-acetate) and EGF treatments (Huang et al. 1999; Ouyang et al. 2005b). In addition, our published studies have shown that arsenite exposure is able to activate PI-3K in mouse epidermal Cl41 cells (Ouyang et al. 2006). To determine the potential involvement of the PI-3K pathway in arsenite-induced HaCat cell transformation, we tested the PI-3K activity in arsenite-exposed HaCat cells. The results showed that the arsenite exposure did increase PI-3K activation in HaCat cells compared with the medium control (Figure 2A, B). We also further confirmed this finding in NHEKs (Figure 2C, D). The aforementioned data demonstrate that PI-3K is implicated in human keratinocyte response to arsenite exposure.

**Figure 3:**
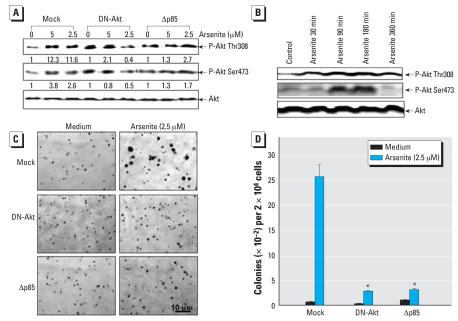
Requirement of the PI-3K/Akt pathway activation for HaCat cell transformation upon arsenite exposure. (*A*) HaCat cells stable transfected with dominant negative mutants of Akt (DN-Akt) or p85 (Δp85) or vector control (Mock) were treated with arsenite in different doses as indicated for 180 min. The number was the relative blots density of phosphorylated Akt compared with total Akt. (*B*) NHEKs were treated with 2.5 μM arsenite at different time points and the phosphorylation of Akt was detected with specific antibodies. (*C,D*) The anchorage-independent growth was evaluated among the HaCat cells stable transfected with vector control, DN-Akt, and Δp85 after repeated exposure to arsenite for 8 weeks. Each bar indicates the mean and SE of triplicate assay wells. *Significant decrease compared with that from HaCat cells transfected with vector (Mock) (*p* < 0.05).

Upon activation, PI-3K generates phosphatidylinositol-3,4,5-trisphosphate (PIP3), a lipid second messenger essential for the translocation of Akt to the plasma membrane where it is phosphorylated and activated by phosphoinositide-dependent kinase-1 (PDK-1) (Alessi et al. 1997; Toker and Cantley 1997). Subsequently, Akt phosphorylates and regulates the function of many downstream cellular proteins involved in the processes of apoptosis, proliferation, and transformation (Alessi et al. 1997; Franke et al. 2003). To test possible Akt activation by arsenite in human keratinocytes, we determined Akt activation in both HaCat and NHEKs by evaluating its phosphorylation at Thr308 and Ser473. The results indicated that arsenite exposure was able to activate Akt in both cells (Figure 3A, B), which was consistent with PI-3K activation. To elucidate the PI-3K/Akt pathway and its role in human keratinocyte response to arsenite response, we established the stable HaCat Δp85 and DN-Akt transfectants. Ectopic expression of Δp85 and DN-Akt dramatically reduced arsenite-induced Akt activation (Figure 3A), and consequently blocked cell transformation upon chronic arsenite exposure in HaCat cells (Figure 3C, D). These results demonstrate the critical role of the PI-3K/Akt pathway in arsenite-induced HaCat transformation.

### Cyclin D1 is a key PI-3K/Akt downstream protein responsible for arsenite-induced transformation of HaCat cells

**Figure 4:**
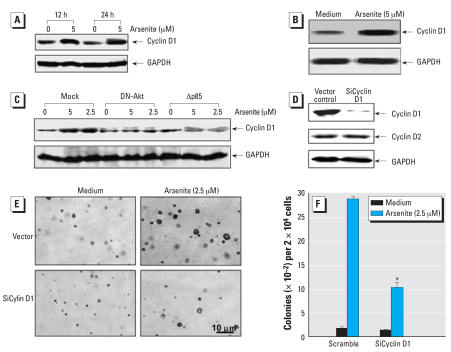
A key role of cyclin D1 in arsenite-induced HaCat cell transformation. HaCat cells (*A*) and NHEKs (*B*) were treated with 5 μM arsenite for the indicated time period (*A*) or for 24 hr (*B*), and the cells were extracted with sample lysis buffer for Western blot analysis to determine cyclin D1 expression. (*C*) HaCat cells stable transfected with vector, DN-Akt, or Δp85, were treated with arsenite at concentrations indicated, and cyclin D1 protein expression levels were evaluated with Western blot analysis. (*D*) Specific knockdown of cylin D1 in HaCat cells was identified with Western blot analysis compared with normal expression of cyclin D2 expression. (*E*,*F*) The capability of anchorage-independent growth activities was compared between cyclin D1 siRNA transfectant and nonspecific control siRNA transfectant after repeatedly treated with arsenite for 8 weeks. Each bar indicates the mean and SE of triplicate assay wells. *Significant decrease compared with that from HaCat cells transfected with control siRNA (Scramble).

It has been thought that the contribution of the PI-3K/Akt pathway to tumorigenesis could be associated with either its regulation of cell apoptosis or cell growth. Our previous studies have shown that arsenite exposure is able to up-regulate cyclin D1 protein expression in HaCat cells, which further mediates cell cycle alternation in HaCat cells (Ouyang et al. 2005b). Thus, it is important to determine whether there is a link between arsenite-induced PI-3K/Akt activation and cyclin D1 protein expression. Arsenite treatment resulted in a marked increase in cyclin D1 protein expression in both HaCat cells (Figure 4A) and NHEKs (Figure 4B), and this cyclin D1 induction was dramatically impaired in Δp85 or DN-Akt stable transfectants (Figure 4C), indicating that the PI-3K/Akt pathway is critical for cyclin D1 protein induction by arsenite. It might be noted that overexpression of DN-Akt was able to block Akt activation, whereas Δp85 only showed a partial inhibition of Akt activation induced upon arsenite treatment (Figure 3A). This differential inhibition of Akt phosphorylation by DN-Akt and Δp85 could be due to the protein expression levels of those two exogenous dominant negative mutants, or alternate pathways may be involved in the Akt activation. It might also be noted that Δp85 is able to block arsenite-induced cyclin D1 expression completely, whereas it shows only partial inhibition on Akt phosphorylation. The explanation for this may be that Akt is only one of p85 downstream kinases, and the other p85 downstream kinases such as protein kinase C, serum gluco-corticoid-inducible kinase, and Rac/CDC42 may also play some role in cyclin D1 protein expression in arsenite responses. In addition, cyclin D1 induction might need PI-3K activation to a certain level, so when Akt activation was relatively low, it was not able to cause cyclin D1 induction. The basal level of Akt phosphorylation in DN-Akt transfectants was higher than that of the vector control (Mock) transfectants. The explanation was that, due to the importance of Akt in normal cell function, the phosphorylation of the endogenous Akt in DN-Akt stable transfectant was elevated to overcome the biological effects caused by over-expression of exogenous DN-Akt. However, the arsenite-induced phosphorylation will be greatly inhibited, as shown in Figure 3A.

To evaluate the contribution of cyclin D1 protein expression to arsenite-induced HaCat cell transformation, we used HaCat cells stably transfected with cyclin D1 siRNA (Ouyang et al. 2005b). As shown in Figure 4D, introduction of cyclin D1 siRNA dramatically reduced the basal level of the cyclin D1 protein expression, whereas it did not affect the basal level of the cyclin D2 protein expression, verifying the specificity of cyclin D1 siRNA. Knockdown of cyclin D1 expression by its siRNA abrogated the HaCat cell transformation induced by arsenite (Figure 4D, E). Collectively, these results indicate that cyclin D1 is not only induced by arsenite exposure through the PI-3K/Akt-dependent pathway but it is also at least one of the key events responsible for arsenite-induced human keratinocyte transformation.

## Discussion

Arsenite is a well-defined human carcinogen, with skin as its primary target organ (Centeno et al. 2002; Huang et al. 2004; Tseng et al. 1968; Yu et al. 2006). Because arsenite has only a weak mutagenic effect, it is thought that its ability to activate some signaling pathways and gene expression responsible for cell growth may play an important role in mediating its carcinogenetic effect (Bernstam and Nriagu 2000). In the present study, we demonstrated that repeated exposure of human keratinocytes to low doses of arsenite resulted in cell transformation with the characteristic of cell anchorage-independent growth in soft agar. The dose we used to repeatedly treat cells did not cause obvious cell death. On the contrary, it promoted cell proliferation as we reported in our recent publication (Ouyang et al. 2007b). The treatment of cells with arsenite also caused the activation of PI-3K/Akt, which thereby plays a critical role in arsenite-induced cell transformation through induction of cyclin D1 expression.

As an important signal pathway for cell survival and growth, PI-3K/Akt has been demonstrated to be associated with tumorigenesis (Nicholson and Anderson 2002; Samuels and Ericson 2006). More than 30% of various solid tumor were found recently to contain mutations in PIK3CA, the catalytic subunit of PI-3K (Samuels and Ericson 2006). The mutation in p85, a regulatory subunit of PI-3K, has also been reported in previous studies (Jimenez et al. 1998; Philp et al. 2001). Recent studies also indicate that Akt is frequently constitutively activated in many types of human cancer (Nicholson and Anderson 2002). Although the mechanisms have not yet been fully characterized, constitutive PI-3K/Akt signaling is believed to promote proliferation and increase cell survival, which is an indispensable event during the process of cancer development (Samuels and Ericson 2006). Current studies demonstrated that arsenite exposure was able to activate PI-3K and Akt, and inhibition of either PI-3K or Akt by their dominant mutants impaired arsenite-induced cell transformation in human skin keratinocytes HaCat, suggesting that the PI-3K/Akt pathway may contribute to arsenite human skin carcinogenic effects.

Reactive oxygen species (ROS) at low concentration may function as a signaling intermediator of cellular responses (Sullivan et al. 1994). The production of ROS in response to arsenite treatment has been observed in various cell lines (Duyndam et al. 2001; Ozaki et al. 2000), suggesting that arsenite may act early in the growth factor signaling pathway. Jung et al. (2003) have clearly demonstrated that the predominant product by arsenite appeared to be hydrogen peroxide (H_2_O_2_) because the arsenite-induced increase in dichlorofluorescein (DCF) fluorescence was completely abolished by pretreatment with catalase but not with heat-inactivated catalase. By eliminating H_2_O_2_ with catalase or *N*-acetylcysteine, they further found that H_2_O_2_ might act as an upstream molecule of PI-3K as well as ERK1/2 (Jung et al. 2003). So we propose that the generation of ROS by arsenite may be associated with various cellular processes, such as PI-3K/Akt pathway activation.

Cyclin D1 could be induced by growth factors and stress, then regulate cell cycle and proliferation (Cook et al. 2000; Perry et al. 1998; Winston and Pledger 1993). Aberrant cyclin D1 expression has been observed early in carcinogenesis (Barnes and Gillett 1998; Fusenig and Boukamp 1998; Weinstein 2000), and overexpression of cyclin D1 was reported in several human cancers, including uterine cervix (Nichols et al. 1996), ovary (Worsley et al. 1997), breast (Michalides et al. 1996), urinary bladder (Proctor et al. 1991), endometrium (Semczuk and Jakowicki 2004), and skin (Rodriguez-Puebla et al. 1999). Antisense to cyclin D1 was reported to inhibit the growth and the tumorigenicity of human colon cancer cells and induce apoptosis in human squamous carcinomas (Arber et al. 1997; Sauter et al. 1999). It has been demonstrated that carcinogenic compounds can induce cyclin D1 expression, which in turn promote tumor cell proliferation (Rodriguez-Puebla et al. 1999; Shen et al. 2006). Our previous studies showed that arsenite could activate the PI-3K/Akt pathway and induce cyclin D1 expression in mouse epidermal cells. In this study, we provided the first direct evidence that cyclin D1 is a downstream target of the PI-3K/Akt signal cascade and involved in the cell transformation caused by arsenite exposure in human keratinocytes.

Although knockdown of cyclin D1 expression by its siRNA markedly inhibited cell transformation of human keratinocytes exposed to arsenite, its effect was less than expression of the dominant negative mutants of PI-3K or Akt (Figure 3D, F). It was likely that some other downstream molecules might also be the PI-3K/Akt downstream targets partially responsible for arsenite-induced cell transformation. For example, in addition to cyclin D1 induction, the PI-3K/Akt pathway has also been reported to mediate the up-regulation of hypoxia-inducible factor 1α and its downstream target gene vascular endothelial growth factor expression (Gao et al. 2004), which has been reported to promote cell transformation, induce the anti-apoptosis genes expression, and subsequently render the cell apoptosis resistance, and promote cell immigration and invasion (Larcher et al. 1998).

In summary, our studies demonstrate that the PI-3K/Akt pathway plays a role in the arsenite-induced transformation of human keratinocytes through the induction of cyclin D1. These results provide novel information for understanding the molecular mechanisms underlying the carcinogenic effect of arsenite on its major target tissue of human skin, which also suggests that the PI-3K/Akt/cyclin D1 pathway might be a target for chemo-prevention of arsenite-induced skin cancer.
